# Supplementary material for: Feedforward and feedback interactions between visual cortical areas use different population activity patterns
Source: Nat Commun. 2022 Mar 1;13:1099. doi: 10.1038/s41467-022-28552-w (PMC8888615; doi:10.1038/s41467-022-28552-w)
Supplement: Supplementary file 1 — Supplementary Information [file 41467_2022_28552_MOESM1_ESM.pdf]

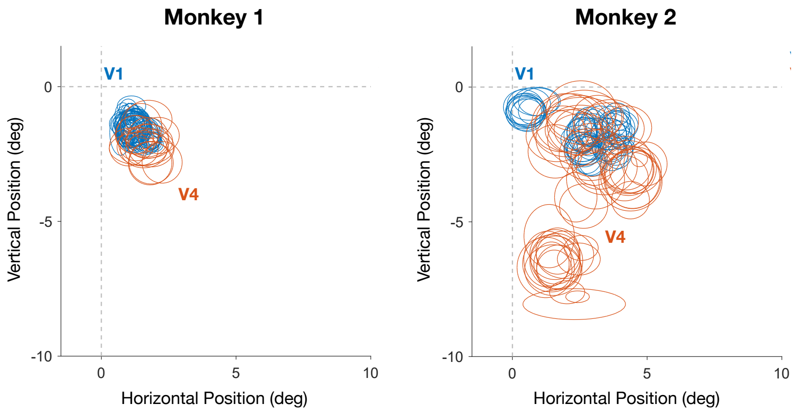

Supplementary Figure 1 Spatial receptive fields for the V1-V4-awake recordings. Lines indicate 60% contour lines of a 2-dimensional Gaussian fit to the receptive fields. Receptive fields were fitted to unsorted multi-unit activity recorded on each channel.

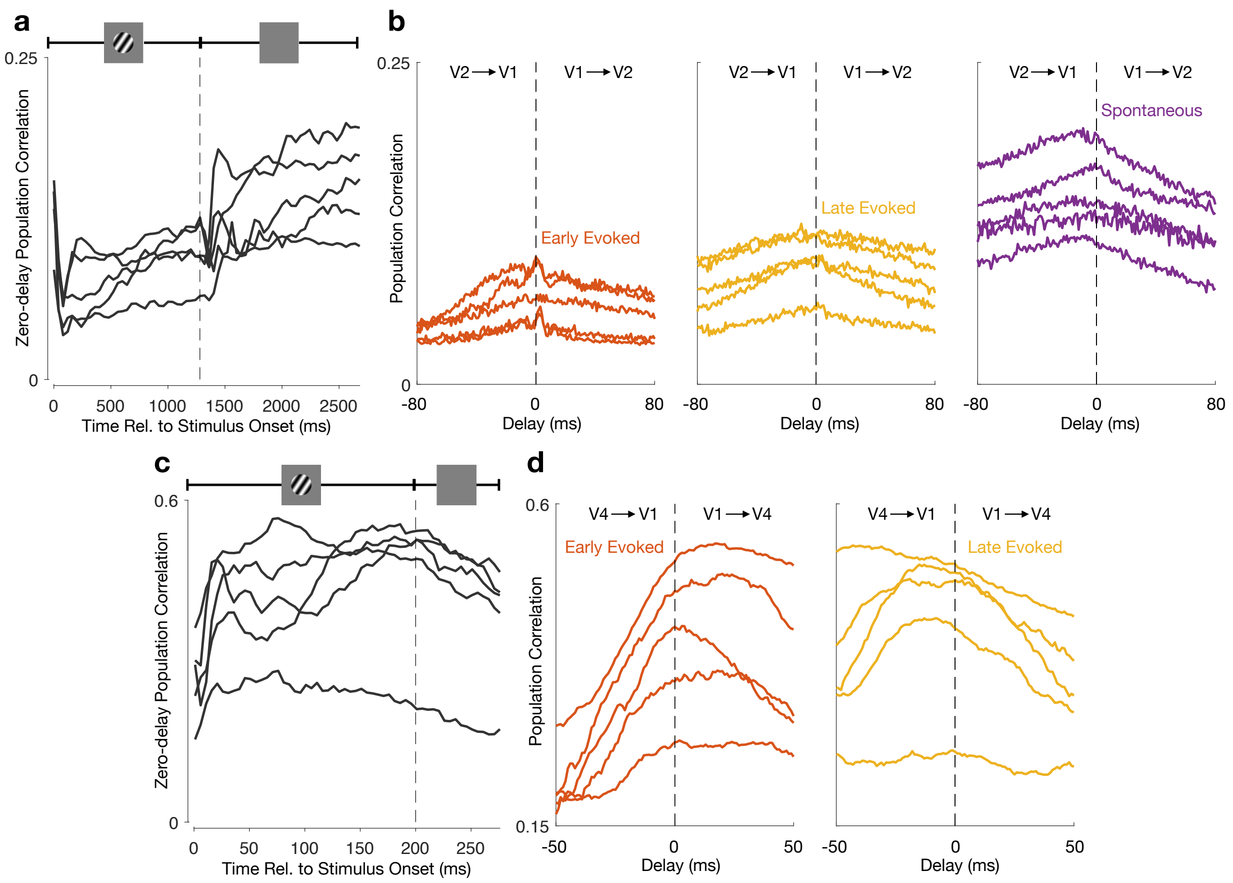

Supplementary Figure 2 Population correlation results are consistent across recording sessions. In all panels, each trace corresponds to one experimental session. **(a)** Same conventions as Fig. 3a. **(b)** Same conventions as Fig. 3b, split into three panels for visual clarity. **(c)** Same conventions as Fig. 4a. **(d)** Same conventions as Fig. 4b, split into three panels for visual clarity.

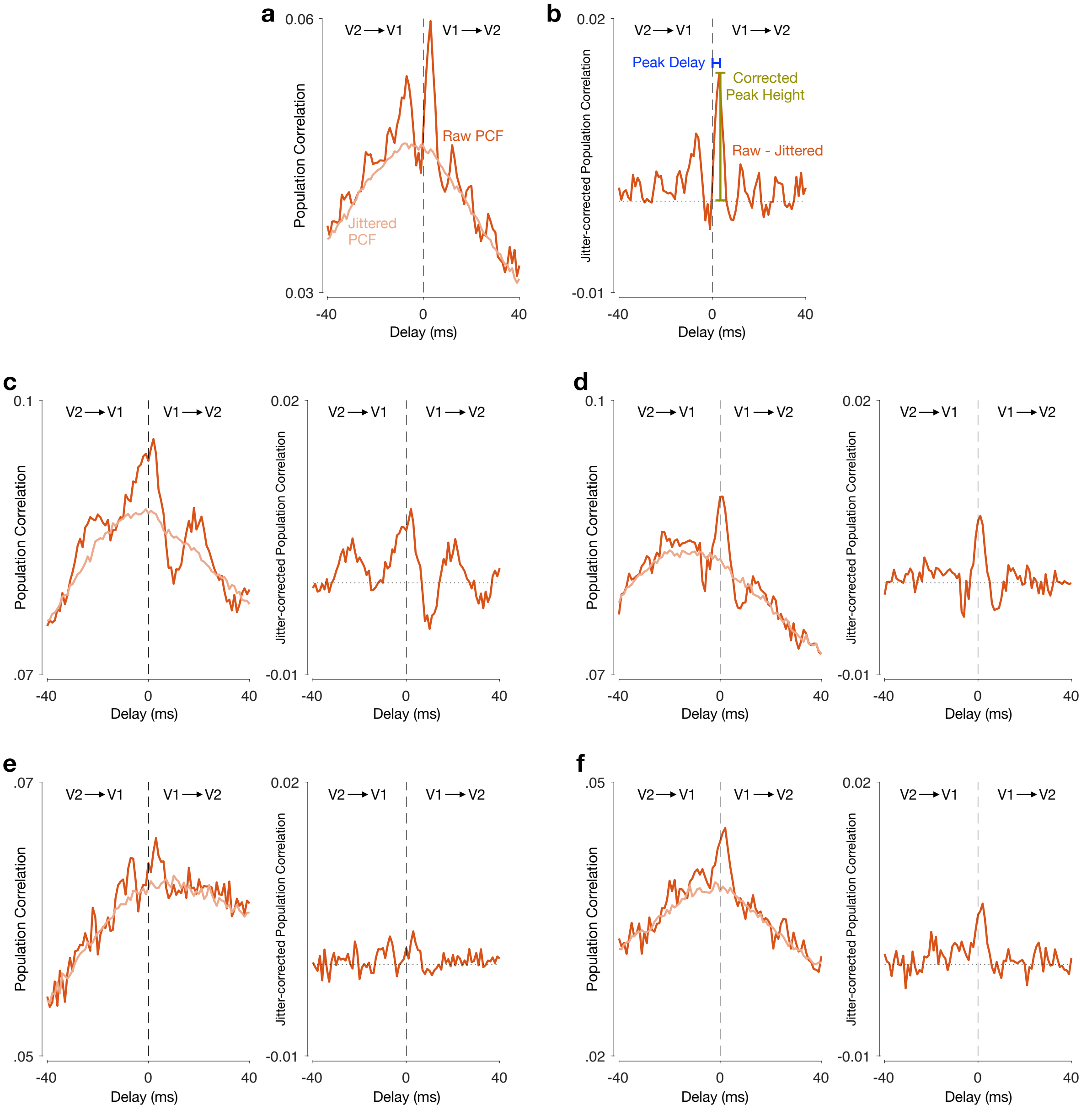

a
0.06
0.03
Population Correlation
V2→V1
V1→V2
Raw PCF
Jittered PCF
-40
0
40
Delay (ms)
b
0.02
-0.01
Jitter-corrected Population Correlation
Peak Delay
Corrected Peak Height
Raw - Jittered
c
0.1
.07
d
e
.07
.05
f
.05
.02

Supplementary Figure 3 Isolating feedforward peaks using a jitter-corrected population correlation function. **(a)** If a feedforward peak is caused by precise spiking coordination across the two areas, it should still be present after the slow-timescale component of the population correlation function is removed. To remove the slow-timescale component, thereby isolating fast-timescale features in the early evoked activity, we computed a jitter-corrected population correlation function[1]. We first computed a jittered population correlation function (Jittered PCF; 25 ms jitter window), as described in ref. 1. We then obtained a jitter-corrected PCF by subtracting the jittered PCF from the PCF based on residual activity (Raw PCF). **(b)** We computed the peak height by finding the maximum value of the jitter-corrected PCF, as well as the corresponding time delay. In this session, a clear peak can be observed at 3 ms. Results across all recording sessions are shown in Fig. 3e. **(c-f)** Population correlation functions for the 4 other recording sessions. Left panels show the Raw PCF (deeper red) and the Jittered PCF (lighter red). Right panels show the jitter-corrected PCF. Note that in two recording sessions the central peak was flanked by secondary peaks. These occurred at different delays for the sessions showing this structure: roughly a 10 ms delay in (a) and 25 ms in (c). There are several possible reasons for this structure, including transient (shared) locking of some neurons to the refresh of the display (100 Hz)[2] and modulation by a weak gamma rhythm (30-80 Hz) induced by the moderate sized, high contrast gratings used to drive responses in V1 and V2[3,4]. In previous work, we have shown that large sinusoidal gratings induce a robust gamma rhythm in these networks[3]. In the experiments of this manuscript, we chose grating size to be sufficient to drive the sampled V1 and V2 neurons. Depending on the scatter of the receptive fields, the size used was sometimes sufficient to have been capable of inducing weak gamma fluctuations[5]. For further assessment of oscillatory interactions between V1 and V2, see Supplementary Fig. 10.

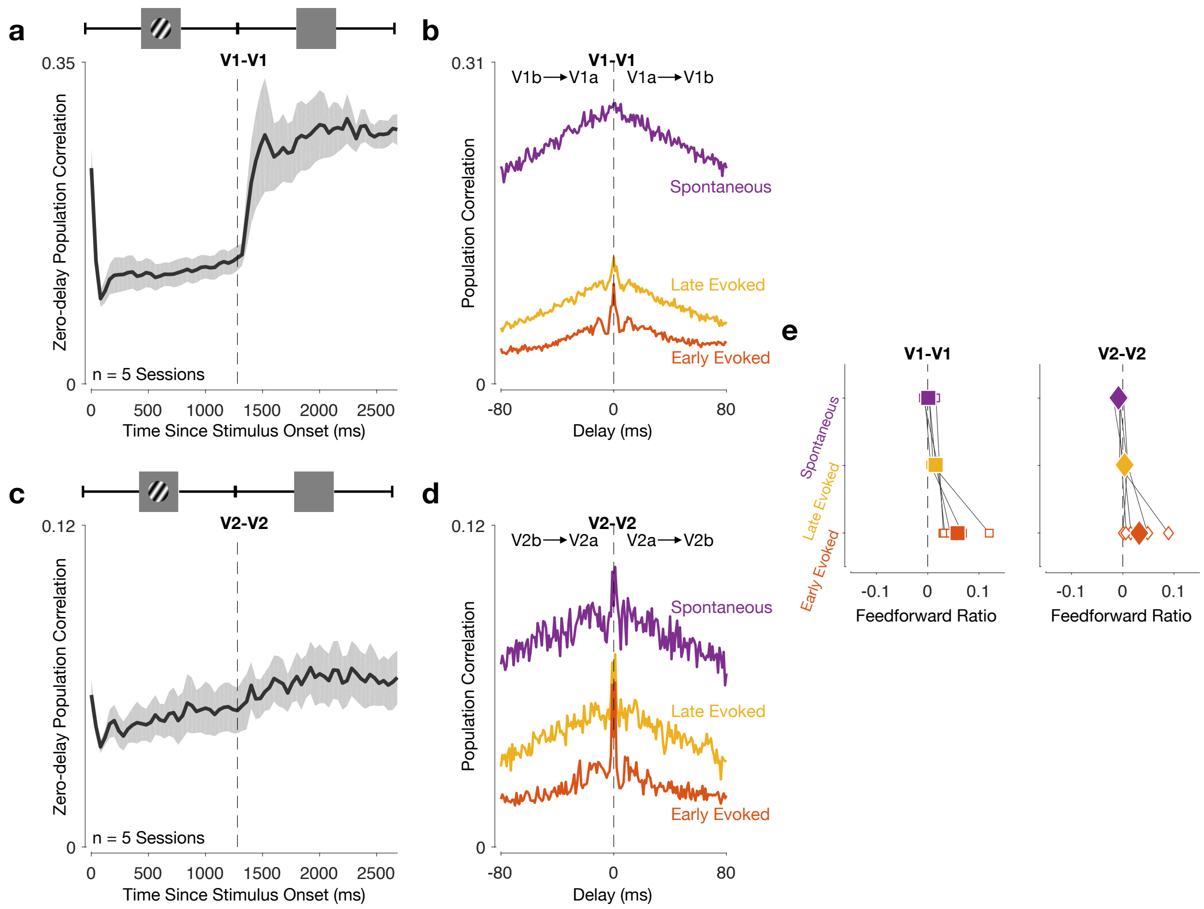

Supplementary Figure 4 Feedforward peak is absent, and feedback-dominated interactions are not evident between subpopulations within V1 or V2. To test whether the effects in Fig. 3 were specific to inter-areal interactions, we randomly divided the neurons in each area into two subpopulations and computed population correlation functions between the subpopulations for each area. **(a)** V1-V1 zero-delay population correlation increases throughout the trial, and is higher for spontaneous activity than for evoked activity. Solid line shows average across all recording sessions. Shading indicates S.E.M. **(b)** V1-V1 population correlation functions for an example session (taken as the average across 10 random divisions into two V1 subpopulations). Same conventions as in Fig. 3b. **(c)** V2-V2 zero-delay population correlation increases throughout the trial, and is higher for spontaneous activity than for evoked activity. Solid line shows average across all recording sessions. Shading indicates S.E.M. **(d)** V2-V2 population correlation function for

an example session (taken as the average across 10 random divisions into two V2 subpopulations). Same conventions as in Fig. 3b. **(e)** There are two key features of inter-areal interactions revealed in Fig. 3 which are not present for within-area interactions. First, the feedforward peaks of the population correlation functions for within-area interactions are centered at 0 ms delay (see panels b and d). This is in contrast to the across-area (V1-V2) case, where there is a feedforward peak shortly after stimulus onset (Fig. 3b,e). Second, within-area interactions (V1-V1, left panel; V2-V2, right panel) were neither feedforward- nor feedback-dominated during spontaneous activity (average spontaneous activity feedforward ratio, computed in the -80 to 80 ms delay range: 0.002 ± 0.004 SEM for V1-V1; -0.007 ± 0.003 SEM for V2-V2; t-test for spontaneous activity feedforward ratio, $p = 0.71$ for V1-V1; $p = 0.09$ for V2-V2. This is in contrast to the across-area (V1-V2) case, where interactions were feedback-dominated during spontaneous activity (Fig. 3d). Note that the feedforward ratio is slightly positive for the early evoked period, although the population correlation functions peak at 0 ms time delay throughout the whole trial. This reflects the slightly greater area under the right half compared to the left half of the population correlation function (panels b and d), likely due to the strong change in correlations at stimulus onset (panels a and c). This effect occurs on a slow timescale and motivates our use of jitter-corrected responses reported in the main text (Fig. 3e). Same conventions as in Fig. 3d.

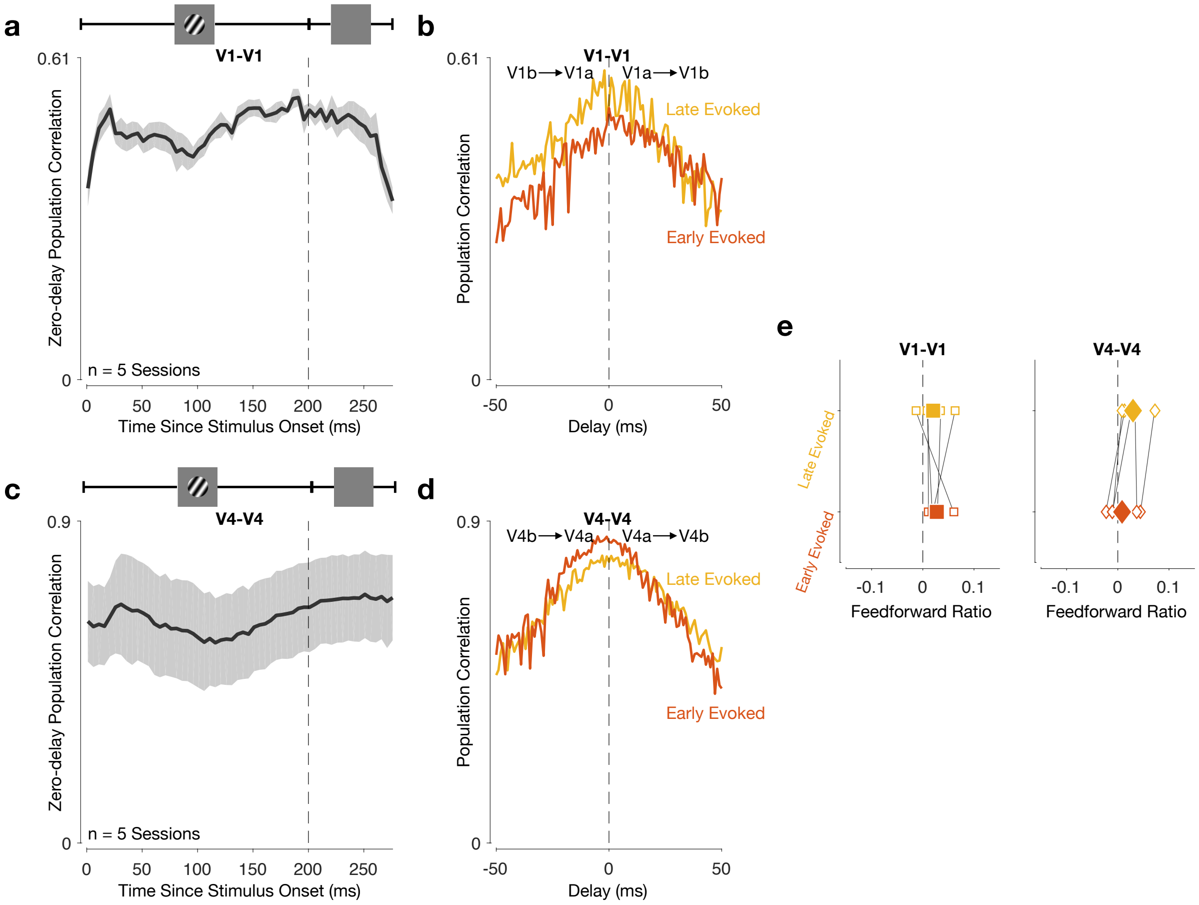

Supplementary Figure 5 Interactions between subpopulations within V1 or V4 recorded in awake animals were neither feedforward- nor feedback-dominated. To test whether the effects in Fig. 4 were specific to inter-areal interactions, we randomly divided the neurons in each area into two subpopulations and computed population correlation functions between the subpopulations for each area. **(a)** V1-V1 zero-delay population correlation is constant throughout the trial. Shading indicates S.E.M. **(b)** V1-V1 population correlation functions for an example session (taken as the average across 25 random divisions into two V1 subpopulations). Same conventions as in Fig. 4b. **(c)** V4-V4 zero-delay population correlation is constant throughout the trial. Solid line shows average across all recording sessions. Shading indicates S.E.M. **(d)** V4-V4 population correlation functions for an example session (taken as the average across 25 random divisions into two V4 subpopulations). Same conventions as in Fig. 4b. **(e)** There are two key features of

inter-areal interactions revealed in Fig. 4 which are not present for within-area interactions. First, the feedforward-dominated interaction shortly after stimulus onset (Fig. 4b,d) is absent here, and the correlation functions are centered at 0 ms delay (see panels b and d). Second, the transition from feedforward- to feedback-dominated interactions in the late evoked period (Fig. 4d) is also absent (average late evoked feedforward ratio, computed in the -50 to 50 ms delay range: 0.027 ± 0.008 SEM for V1-V1; 0.031 ± 0.011 SEM for V4-V4; one-sided paired Wilcoxon signed-rank test for difference between early evoked and late evoked activity across all 5 recording sessions, $p = 0.41$ for V1-V1; $p = 0.97$ for V4-V4). Same conventions as in Fig. 4d.

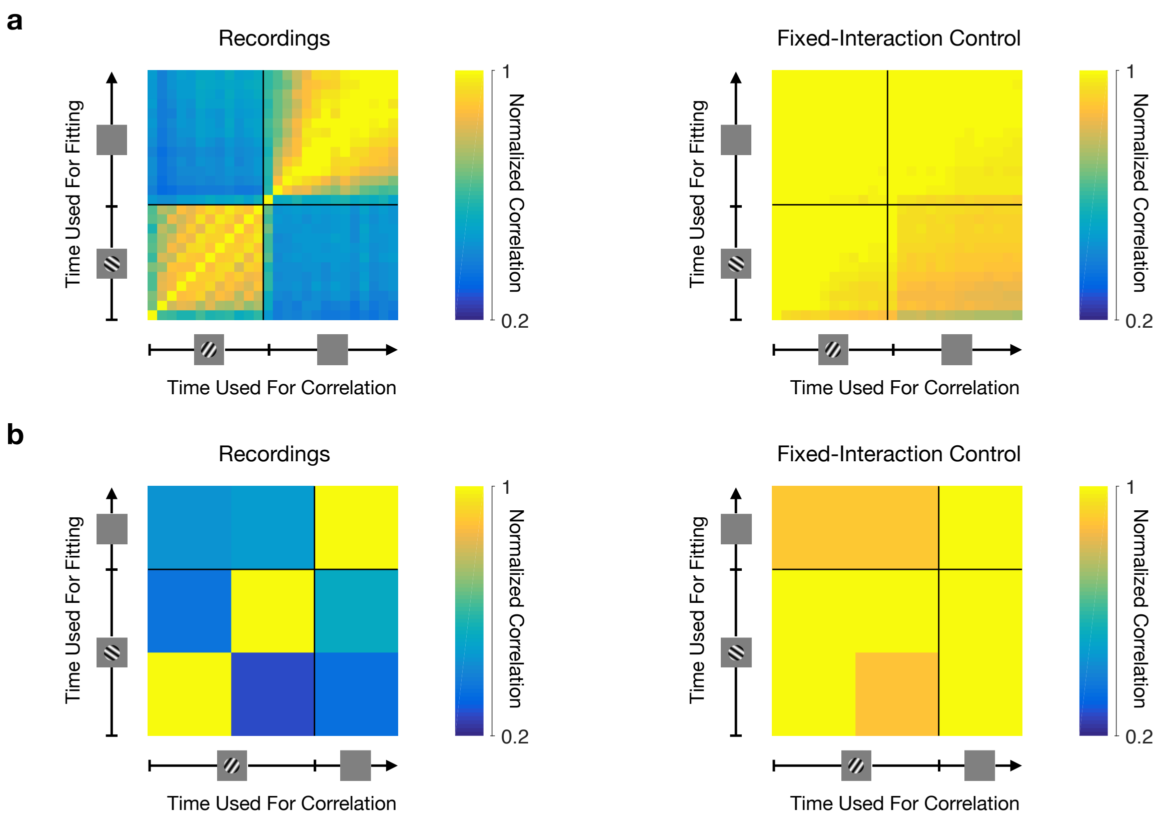

Supplementary Figure 6 Ensuring that changes in activity patterns most related across areas cannot be ascribed to changes in the within-area population covariance structure. **(a)** We generated V1-V2 surrogate data that had approximately the same within-area covariance structure as the recorded data for each epoch, but for which the inter-areal interaction structure was held fixed (see Methods and Supplementary Note). For this synthetic data, our analysis identified a stable interaction structure (right, compare to left reproduced from Fig. 6d which is based on recorded activity). Same conventions as in Fig. 6d. **(b)** The same was true for the V1-V4 interactions (right, compare to left reproduced from Fig. 6e which is based on recorded activity). Same conventions as in Fig. 6e.

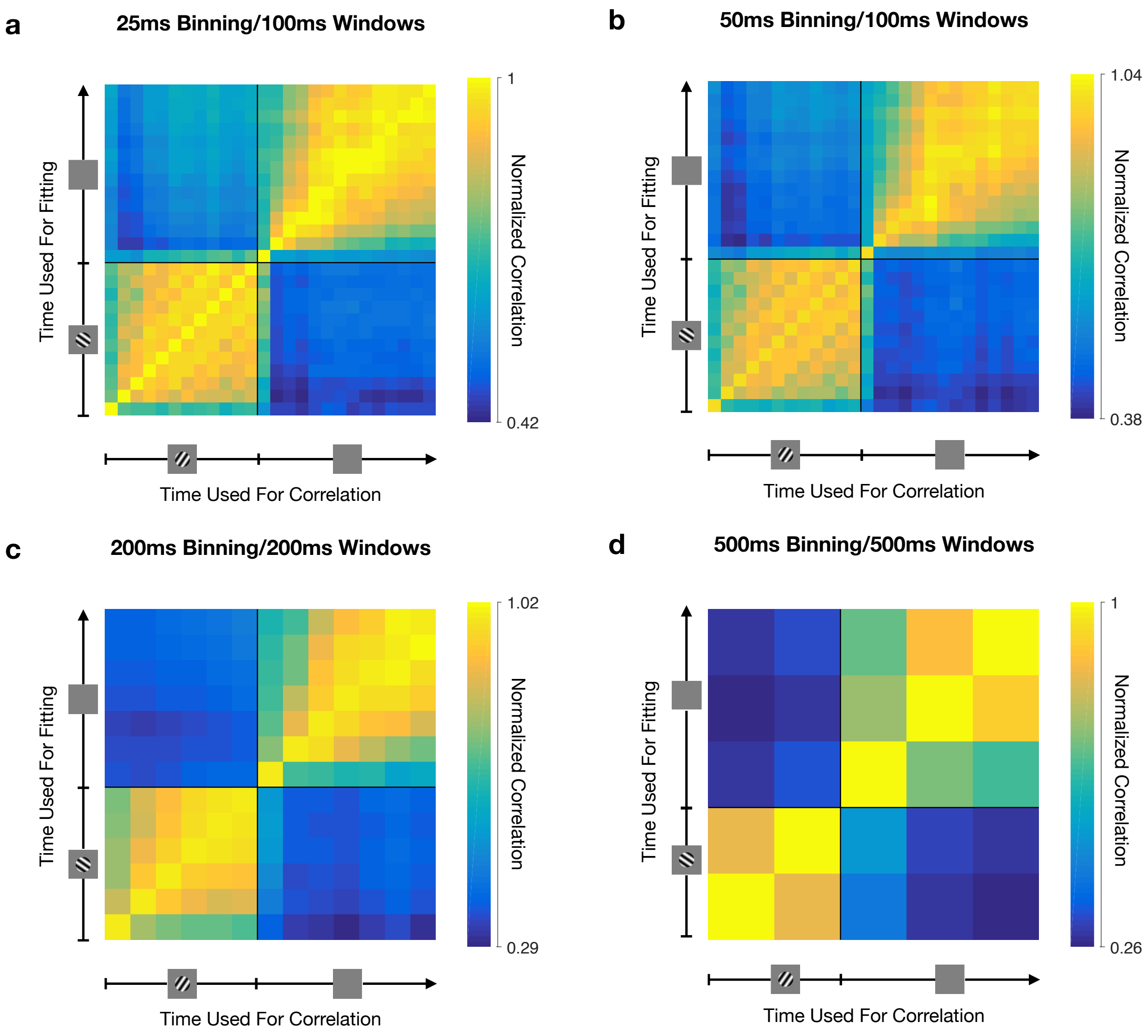

Supplementary Figure 7 Changes in interaction structure are robust to the time-scale considered. To assess the timescales of the correlated activity across areas, we repeated the analysis of Fig. 6 while varying the size of the spike count bins and the duration of the temporal windows analyzed on each trial. When the spike count bins are large (e.g., one bin per trial), the analysis emphasizes the slow timescale correlations across areas. In contrast, when the spike count bins are small (multiple bins per trial), the analysis focuses on fast timescale correlations. The number of bins per trial is equal to the duration of the temporal window divided by the spike count bin size. The approach of assessing timescales by varying the spike count bin width has been used previously to characterize

the timescale of pairwise correlations[1,6,7]. **(a-d)** We found the results shown in Fig. 6 are robust to the spike count bin size and the temporal window duration. All panels follow the same conventions and analysis settings as in Fig. 6d, with the exception of the spike count bin size and duration of the temporal window during which bins are aggregated. In Fig. 6, we used 100 ms spike count bins and a 100 ms temporal window (1 bin per trial).

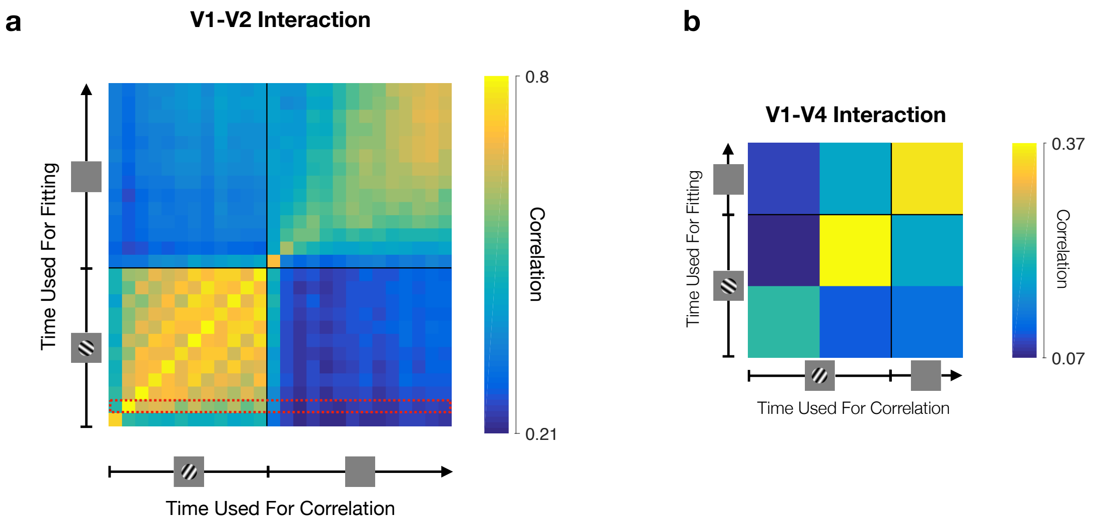

Supplementary Figure 8 Feedforward canonical dimensions capture little correlation during the feedback-dominated period. We asked whether the canonical dimensions identified during the feedforward-dominated period remain active during the feedback-dominated period. In Fig. 6, we showed that feedforward dimensions only capture a fraction of inter-area correlations in the feedback period (using normalized correlation values). It is possible that they still account for a large absolute correlation value, but lower than that for the dimensions identified during the feedback period. The analysis shown here indicates that that is not the case: the amount of inter-areal correlations captured by dimensions fit during the feedforward dominated period drops off sharply during the feedback-dominated period. This is true for both the **(a)** V1-V2 and **(b)** V1-V4 recordings. Panels (a) and (b) follow the same conventions as Fig. 6d and Fig. 6e, respectively, but show raw canonical correlation instead of normalized correlation. The results are slightly different from those shown in Fig. 3a and Fig. 3b due to the different spike count bin width and temporal window duration used in those analyses. As in Fig 6, the correlation values here are cross-validated.

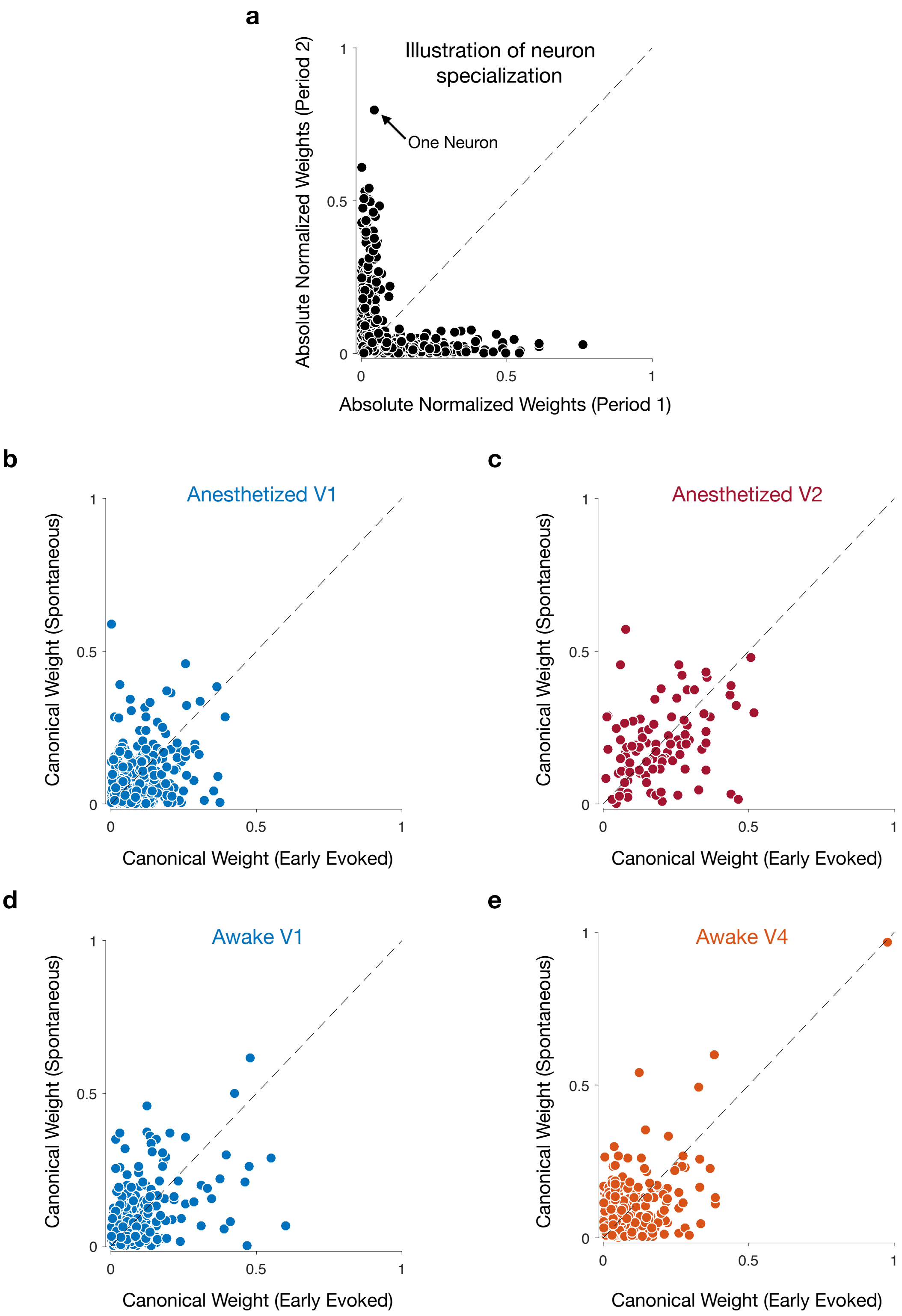
a
Illustration of neuron specialization
One Neuron
Absolute Normalized Weights (Period 2)
Absolute Normalized Weights (Period 1)
0
0.5
1
b
Anesthetized V1
c
Anesthetized V2
d
Awake V1
e
Awake V4
Canonical Weight (Spontaneous)
Canonical Weight (Early Evoked)

Supplementary Figure 9 The same neurons are involved in both feedforward and feedback interactions. **(a)** One possibility is that the change in interaction structure we observed in Fig. 6 results from some neurons being exclusively involved in feedforward interactions, and other neurons being exclusively involved in feedback interactions. If this were the case, we would find that the canonical weights for some neurons would be large during period 1 and small during period 2, and those for other neurons would be large during period 2 and small during period 1. For each neuron, we can plot its absolute canonical weight during one period versus its absolute canonical weight during another period (each dot corresponds to one neuron). The weights are normalized such that the canonical dimensions have norm 1. **(b)** For the anesthetized V1 populations, most neurons are involved in both feedforward (early evoked) and feedback (spontaneous) interactions. This is seen by the lack clustering of neurons along the axes, in contrast to the scenario illustrated panel (a). The same was true for **(c)** the anesthetized V2 populations, **(d)** awake V1 populations, and **(e)** awake V4 populations.

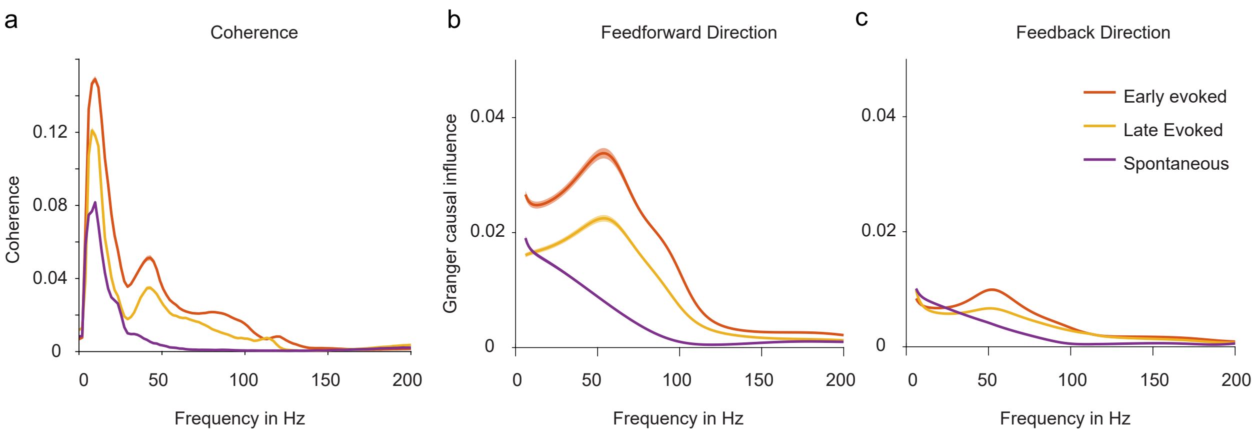

Supplementary Figure 10

Much previous work on inter-areal signaling has been performed using local field potentials (LFPs)[8–13]. Unlike spiking activity, LFPs are not propagated between areas. Nevertheless, LFPs provide some measure of population activity, and analyzing the relationship between LFPs recorded in different brain areas has been used to infer the communication between areas. Of particular interest in the context of this study, recent work has suggested that feedforward and feedback signaling are reflected in different frequency bands of the LFP – gamma (30-80 Hz) and alpha/beta, respectively[10,13,14]. To understand the relationship between our population spike-based measurements and this prior work, we analyzed the local field potentials which we had recorded simultaneously with the spiking activity.

LFP data were obtained by low-pass filtering (0.5-200 Hz or 0.3-250 Hz) the raw voltage signal on each electrode and digitizing at 1 kHZ. Notch filters were used on an electrode-by-electrode basis to remove line noise. Trials with artifacts were excluded from further analysis, using the criteria of ref. 15. We also removed the trial averaged event-related potential so that this component of the signal would not dominate the low frequency components of analysis[13]. Both coherence and spectral Granger causality were computed between all V1-V2 electrode pairs, in the same 5 recording sessions which yielded the spiking data analyzed in the main text (n=3220 pairs). For both coherence and

Granger analysis we defined three 512 ms response epochs: early evoked, beginning 50 ms after stimulus onset (a period that encompasses the early evoked period used for spiking data); late evoked, beginning 512 ms before stimulus offset (a period that encompasses the late evoked period used for spiking data); and spontaneous, beginning 512 ms before visual stimulus onset (encompassing the spontaneous epoch used for spiking data). Coherence was calculated using the multi- taper method (3 tapers) of the Chronux toolbox[16]. Spectral Granger causality was estimated using the MVGC toolbox[17]. In a subset of the data, we varied model order systematically and found an order of 8 (i.e., 8 samples, or 8 ms) yielded the best performance on average; we therefore used this order for all V1-V2 electrode pairs. Our results did not vary significantly when using other values, up to 50. We did not analyze LFPs in the V1-V4 recordings because the brief stimulus presentation yielded poor frequency resolution for the different response epochs.

(a) We first assessed V1-V2 LFP coherence in the early evoked, late evoked, and spontaneous epochs. Coherence between V1 and V2 LFPs displayed two prominent peaks: one centered at 9 Hz (in the alpha range) and another at 43 Hz (in the gamma range). The gamma band coherence was strongest in the early evoked period, weaker in the late evoked period, and absent in the spontaneous epoch. If one assumes that gamma coherence is indicative of feedforward signaling, then this pattern of results is consistent with our spike-based analyses which also suggested a gradual weakening of feedforward signaling during evoked activity and little evidence of feedforward signaling during spontaneous activity. In the alpha frequency range, the LFP coherence was also strongest in the early evoked period and weakest during spontaneous activity. If alpha coherence is indicative of feedback signaling, this is different from the signaling indicated by population spiking activity. Specifically, our spike-based analyses would suggest that alpha coherence should be strongest, not weakest, during the spontaneous epoch.

(b,c) We next turned to spectral Granger causality analysis. We fit Granger models predicting V2 LFPs using V1 LFPs (the feedforward direction; panel b) and predicting V1 LFPs using V2 LFPs (the feedback direction; panel c). In the feedforward models, Granger influence was highest in the gamma frequencies and, in that range, most prominent during early evoked period and weakest during spontaneous activity. There was no Granger influence peak in the alpha or beta bands for feedforward V1-V2 models. Under the proposal that gamma band activity is related to feedforward signaling, these results are consistent with our analysis of population spiking activity, in which feedforward signaling was most evident in the early evoked period and least evident during spontaneous activity. In feedback models, the Granger influence resembled that evident in the feedforward model, but with lower influence values. That gamma influence is stronger in the feedforward than feedback model is consistent with prior work (see below). But the absence of an alpha or beta peak in the feedback direction is unexpected, given the directionality of inter-areal signaling suggested by our spiking data.

In summary, LFP-based analysis yielded both similarities and differences with the inferences drawn from population spike-based analysis. There is good qualitative agreement between the strength of feedforward signaling suggested by spiking activity and by coherence and Granger analyses of gamma- band LFP activity. In epochs when spiking activity suggested stronger feedforward signaling, both coherence and Granger feedforward influence in the gamma band were elevated. However, the strong feedback signaling evident in spiking activity – particularly during spontaneous activity – was not consistently evident in alpha or beta components of the LFP. Granger analysis in the feedback direction did not reveal a peak in these frequencies. We did observe a peak in the alpha range in coherence – and during spontaneous activity coherence was strongest at alpha frequencies – but coherence was stronger still during evoked activity.

Our LFP results are broadly consistent with previous studies of inter-areal LFP coherence and Granger influence in macaque visual cortex. For instance, previous V1-V2[14] and V1-V4[10,14,18] studies have found elevated coherence in alpha/beta and gamma frequency ranges. Granger models consistently reveal strong influence (relative to other frequencies) in gamma frequencies, in the feedforward direction (e.g., V1-V4 in ref. 14 and ref. 18; and V1-V2 in ref. 14). Influence in the gamma range is weaker in feedback models[10,14,18], as we also observe in our LFP data. Some studies have found Granger influence to be elevated in the alpha/beta band as well, particularly in feedback models[10,14,13]. But ref. 18, like us, did not find a strong alpha/beta component (their Figure 3).

Detailed comparisons across studies are complicated by differences in recordings methodology (LFP recorded using Utah arrays and tetrodes in specific targeted cortical layers in our case, compared to surface ECoG recordings in ref. 14 and ref. 18, and laminar probe recordings in ref. 10), pre-processing (e.g. whether the signal is referenced to the activity at nearby recordings, as in the cited ECoG studies), and details of the analysis (the MVGC toolbox in our case and ref. 10, compared to nonparametric spectral matrix factorization in ref. 14 and ref. 18). In this regard, the results presented here are not meant as an exhaustive comparison to this prior work.

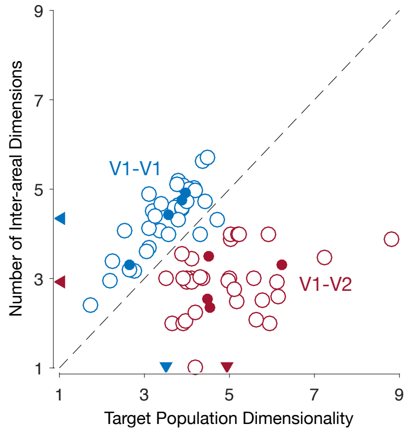

Supplementary Figure 11 A communication subspace is evident when using Canonical Correlation Analysis (CCA) to characterize inter-areal interactions. We previously reported that the interaction between V1 and V2 was low dimensional (termed a communication subspace) using Reduced-Rank Regression (RRR)[19]. RRR is closely related to Canonical Correlation Analysis (CCA), which we employed in this work (for a review, see ref. 20). One might wonder whether CCA also identifies a communication subspace between V1 and V2. We repeated the analysis in our previous work, using the same data that was analyzed there[19], but using CCA instead of RRR to relate the activity across areas. To determine the number of dimensions involved in inter-areal interactions, we first identified the number of canonical dimensions that yielded the highest cross-validated data likelihood for a probabilistic CCA (pCCA) model. We then fit a pCCA model with the corresponding dimensionality using all trials, and computed the associated inter-areal covariance matrix. Finally, we used Singular Value Decomposition (SVD) to determine the smallest number of dimensions that captured 95% of the inter-areal covariance, and used that number as our estimate of the number inter-areal dimensions.

As in our previous work[19], we found that fewer dimensions were required to characterize inter-areal interactions (V1-V2; red triangle on vertical axis) than within-area interactions (V1-V1; blue triangle on vertical axis). In contrast to Fig. 3, where we identified a single

significant canonical pair for each epoch and time delay, here we identify around 3 dimensions of inter-areal interaction. This is largely due to the larger binning windows used here (100 ms vs. 1 ms in Fig. 3). Importantly, the lower number of dimensions required to account for inter-areal interactions, compared to within-area interactions, was not a result of lower dimensional activity in the V2 population, as the population activity dimensionality was higher in V2 than in the held-out V1 populations (compare blue and red triangles on horizontal axis). Moreover, the number of predictive dimensions identified by RRR was highly correlated with the number of canonical dimensions identified by CCA (Pearson correlation coefficient $r^2 = 0.89$ across all datasets; not shown). Open circles corresponds to each dataset, solid circles denote mean across datasets for each recording session. Triangles denote mean across all recording sessions.

Supplementary Note

Characterizing changes in the interaction structure

What constitutes a change in the interaction structure? In other words, how can we evaluate whether or not different activity patterns are involved in inter-areal interactions during different trial epochs? Using Canonical Correlation Analysis (CCA, see Methods) to characterize inter-areal interactions, one might wonder whether changes in the canonical dimensions across two epochs are a good indication of a change in the interaction structure. Here, we show that directly levering the canonical dimensions to test for changes in the interaction structure can be misleading, and propose an alternative approach based on the probabilistic CCA (pCCA) model[21].

Suppose we identify q pairs of canonical dimensions, and represent them as the columns of matrices $\mathbf{A}_q$ and $\mathbf{B}_q$, which have dimensions $p_x \times q$ and $p_y \times q$, respectively, where p_x and p_y are the number of recorded neurons in each of the two areas. The column space of each matrix defines a subspace in each area within which activity is most correlated across areas. If one seeks to compare the canonical dimensions identified during two trial epochs, one possibility is to compare the column spaces of matrices $\mathbf{A}_q$ and $\mathbf{B}_q$ for two different epochs.

There is, however, a potential problem with using this approach to ask whether there was a meaningful change in the inter-areal interaction structure. We can illustrate this issue by considering data generated from a pCCA model. pCCA is defined by the following equations:

$$\mathbf{z} \sim \mathcal{N}(\mathbf{0}, \mathbf{I}_q) \tag{1}$$

$$\mathbf{x}|\mathbf{z} \sim \mathcal{N}(\mathbf{W}_x\mathbf{z}, \mathbf{\Psi}_x) \tag{2}$$

$$\mathbf{y}|\mathbf{z} \sim \mathcal{N}(\mathbf{W}_y\mathbf{z}, \mathbf{\Psi}_y) \tag{3}$$

where $\mathbf{z}$ is a $q \times 1$ latent variable, $\mathbf{x}$ and $\mathbf{y}$ correspond to the neuronal activity recorded in each of two cortical areas, with dimensionalities $p_x \times 1$ and $p_y \times 1$, respectively, p_x and p_y are the number of neurons recorded in each area, and $q \leq \min(p_x, p_y)$. The identity matrix $\mathbf{I}_q$ has dimensions $q \times q$. The mapping matrices $\mathbf{W}_x$ and $\mathbf{W}_y$ have dimensions $p_x \times q$ and $p_y \times q$, respectively. The covariance matrices $\mathbf{\Psi}_x$ and $\mathbf{\Psi}_y$ have dimensions $p_x \times p_x$ and $p_y \times p_y$, respectively. We assume, without loss of generality, that $\mathbf{x}$ and $\mathbf{y}$ are mean-centered. CCA and pCCA return the same correlation values, so both methods result in the same population correlation functions. The advantage of pCCA here is that it provides us with a more complete description of the fitted model and its underlying assumptions.

According to pCCA's graphical model (Supplementary Fig. 12a), we can describe the observed activity in each area as having an "across-area" component and a "within-area" component. The across-area component emerges via the linear mapping between the shared latent variable $\mathbf{z}$ and each observed variable, $\mathbf{x}$ and $\mathbf{y}$. This mapping is defined by the matrices $\mathbf{W}_x$ and $\mathbf{W}_y$. The within-area components are defined to be Gaussian with unconstrained covariance matrices $\mathbf{\Psi}_x$ and $\mathbf{\Psi}_y$.

The relationship between the column spaces of the matrices $\mathbf{A}_q$ and $\mathbf{B}_q$ computed by classical CCA and the parameters of the pCCA model is given by:

$$\bar{\mathbf{A}}_q = \mathbf{\Sigma}_{xx}^{-1}\mathbf{W}_x \tag{4}$$

$$= \left(\mathbf{W}_x{\mathbf{W}_x}^T + \mathbf{\Psi}_x\right)^{-1}\mathbf{W}_x \tag{5}$$

$$\bar{\mathbf{B}}_q = \mathbf{\Sigma}_{yy}^{-1}\mathbf{W}_y \tag{6}$$

$$= \left(\mathbf{W}_y{\mathbf{W}_y}^T + \mathbf{\Psi}_y\right)^{-1}\mathbf{W}_y \tag{7}$$

where $\bar{\mathbf{A}}_q$ and $\bar{\mathbf{B}}_q$ have the same column space as $\mathbf{A}_q$ and $\mathbf{B}_q$, respectively (see the "Relationship between CCA and pCCA" section below). This shows that the subspaces spanned by the canonical dimensions in each area depend on the within-area noise parameters $\mathbf{\Psi}_x$ and $\mathbf{\Psi}_y$. Thus, changes to the within-area components lead to changes in the subspaces spanned by the canonical dimensions, even if the across-area components remain fixed. Measuring changes in the interaction structure by measuring to what extent the subspaces spanned by the canonical dimensions differ would thus lead us to conclude that across-area interaction structure had changed, even though only the within-area components were altered.

We can gain further intuition into the pCCA model by inspecting the joint covariance matrix (Supplementary Fig. 12b) The covariance for each area, $\mathbf{\Sigma}_{xx}$ ($\mathbf{\Sigma}_{yy}$), is composed of an across-area component, $\mathbf{W}_x{\mathbf{W}_x}^T$ (resp. $\mathbf{W}_y{\mathbf{W}_y}^T$) and within-area component, $\mathbf{\Psi}_x$ (resp. $\mathbf{\Psi}_y$). Supplementary Figure 12c illustrates this covariance decomposition for one of the areas (ellipses represent each covariance component). For the across area covariance, however, we have $\mathbf{\Sigma}_{xy} = \mathbf{W}_x{\mathbf{W}_y}^T = \mathbf{\Sigma}_{yx}^T$. Thus, the across-area covariance structure is solely determined by the linear mapping matrices $\mathbf{W}_x$ and $\mathbf{W}_y$.

Given that the across-area component in the pCCA model is solely determined by the mapping matrices $\mathbf{W}_x$ and $\mathbf{W}_y$, we can quantify changes in the interaction structure by comparing those matrices for different trial epochs. We will take this approach, and use a

pCCA model to estimate the $\mathbf{W}_x$ and $\mathbf{W}_y$ matrices, and in turn use changes in these matrices to detect changes in the interaction structure. We need to first define how to measure differences between the $\mathbf{W}_x$ and $\mathbf{W}_y$ matrices estimated at different times during the trial. As mentioned above, $\mathbf{W}_x$ and $\mathbf{W}_y$ are underdetermined, so an element by element comparison (e.g., the Frobenius norm of the difference between two $\mathbf{W}_x$ matrices fit at different epochs in the trial) is not suitable. We defined our difference metric to be based on differences between the column spaces of $\mathbf{W}_x$ and $\mathbf{W}_y$, i.e., our measure of how much the interaction structure changes across different epochs is only sensitive to changes in the subspaces spanned by the dimensions along which activity is related across areas. To be conservative, we will not consider scaling and affine transformations of these dimensions (which do not change the subspace spanned by these dimensions) as changes to the interaction structure, although they might reflect interesting changes for other analysis goals.

Specifically, we will measure differences between the column spaces of $\mathbf{W}_x$ and $\mathbf{W}_y$ by comparing the inter-area correlation these subspaces account for. To compare the interaction structure identified during two epochs in the trial, indexed by t and t', we first fit a pCCA model at each epoch, yielding parameters $\theta^t = \{\mathbf{W}_x^t, \mathbf{W}_y^t, \mathbf{\Psi}_x^t, \mathbf{\Psi}_y^t\}$ and $\theta^{t'} = \{\mathbf{W}_x^{t'}, \mathbf{W}_y^{t'}, \mathbf{\Psi}_x^{t'}, \mathbf{\Psi}_y^{t'}\}$. We then ask: given the observed (sample) within-area covariance matrices at time t', $\mathbf{\Sigma}_{xx}^{t'}$ and $\mathbf{\Sigma}_{yy}^{t'}$, how correlated would the activity across areas be if instead of the estimated matrices $\mathbf{W}_x^{t'}$ and $\mathbf{W}_y^{t'}$, the interaction was instead described by matrices $\mathbf{W}_x^t$ and $\mathbf{W}_y^t$? More specifically, how much do across-area correlations change if we replace the column space of $\mathbf{W}_x^{t'}$ and $\mathbf{W}_y^{t'}$ by the column space of $\mathbf{W}_x^t$ and $\mathbf{W}_y^t$? We can use equations 4 and 6 to compute the subspace spanned by the canonical dimensions

induced by $\mathbf{W}_x^t$ and $\mathbf{W}_y^t$ (see the "Relationship between CCA and pCCA" section below):

$$\bar{\mathbf{A}}_q^{t',t} = \mathbf{\Sigma}_{xx}^{t'\ -1}\mathbf{W}_x^t \tag{8}$$

$$\bar{\mathbf{B}}_q^{t',t} = \mathbf{\Sigma}_{yy}^{t'\ -1}\mathbf{W}_y^t \tag{9}$$

and measure the amount of across-area correlation captured by $\bar{\mathbf{A}}_q^{t',t}$ and $\bar{\mathbf{B}}_q^{t',t}$. We then compare that amount of across-area correlation to the correlation that would have resulted from using $\mathbf{W}_x^t$ and $\mathbf{W}_y^t$ (i.e., the across-area correlation captured by $\bar{\mathbf{A}}_q^{t',t'}$ and $\bar{\mathbf{B}}_q^{t',t'}$). The results of this analysis are shown in Fig. 6. Note that both $\bar{\mathbf{A}}_q^{t',t}$ and $\bar{\mathbf{A}}_q^{t',t'}$ (resp. $\bar{\mathbf{B}}_q^{t',t}$ and $\bar{\mathbf{B}}_q^{t',t'}$) are computed using the same covariance matrix $\mathbf{\Sigma}_{xx}^{t'}$ (resp. $\mathbf{\Sigma}_{yy}^{t'}$). Thus, any differences between $\bar{\mathbf{A}}_q^{t',t}$ and $\bar{\mathbf{A}}_q^{t',t'}$ (resp. $\bar{\mathbf{B}}_q^{t',t}$ and $\bar{\mathbf{B}}_q^{t',t'}$) are the result of differences between $\mathbf{W}_x^t$ and $\mathbf{W}_x^{t'}$ (resp. $\mathbf{W}_y^t$ and $\mathbf{W}_y^{t'}$). Specifically, differences between the column spaces of $\bar{\mathbf{A}}_q^{t',t}$ and $\bar{\mathbf{A}}_q^{t',t'}$ (resp. $\bar{\mathbf{B}}_q^{t',t}$ and $\bar{\mathbf{B}}_q^{t',t'}$) are the result of differences between the column spaces if $\mathbf{W}_x^t$ and $\mathbf{W}_x^{t'}$ (resp. $\mathbf{W}_y^t$ and $\mathbf{W}_y^{t'}$; see "Relationship between CCA and pCCA" section below). Supplementary Table 1 describes this process in detail.

Supplementary Table 1: Comparing inter-area interaction structure across time

Result: Normalized correlations $\zeta_q^{t',t}$ for all t' and t, and for all choices of q

Given sets of observations $\{\mathbf{X}^t, \mathbf{Y}^t\}_{train}$ and $\{\mathbf{X}^t, \mathbf{Y}^t\}_{test}$, for each trial epoch t

for $q = 1, ..., \min(p_x, p_y)$ **do**

  for $\forall t$ **do**

    Fit pCCA with latent dimensionality q to the training set $\{\mathbf{X}^t, \mathbf{Y}^t\}_{train}$

     yielding: $\theta^t = \{\mathbf{W}_x^t, \mathbf{W}_y^t, \mathbf{\Psi}_x^t, \mathbf{\Psi}_y^t\}$

    for $\forall t'$ **do**

      Compute across-area correlation in the test set $\{\mathbf{X}^{t'}, \mathbf{Y}^{t'}\}_{test}$:

1. Compute correlation subspaces $\bar{\mathbf{A}}^{t',t}$ and $\bar{\mathbf{B}}^{t',t}$:

   $\bar{\mathbf{A}}^{t',t} = {\mathbf{\Sigma}_{xx}^{t'}}^{-1}\mathbf{W}_x^t$ and $\bar{\mathbf{B}}^{t',t} = {\mathbf{\Sigma}_{yy}^{t'}}^{-1}\mathbf{W}_y^t$

   where $\mathbf{\Sigma}_{xx}^{t'}$ and $\mathbf{\Sigma}_{yy}^{t'}$ are computed using $\{\mathbf{X}^{t'}, \mathbf{Y}^{t'}\}_{test}$

2. Project the $\{\mathbf{X}^{t'}, \mathbf{Y}^{t'}\}_{test}$ onto $\bar{\mathbf{A}}^{t',t}$ and $\bar{\mathbf{B}}^{t',t}$:

   $\hat{\mathbf{X}}^{t'} = \mathbf{X}^{t'}\bar{\mathbf{A}}^{t',t}$ and $\hat{\mathbf{Y}}^{t'} = \mathbf{Y}^{t'}\bar{\mathbf{B}}^{t',t}$

3. Apply CCA to $\{\hat{\mathbf{X}}^{t'}, \hat{\mathbf{Y}}^{t'}\}$ and sum all q canonical correlations,

   obtaining $r_q^{t',t}$

    end

  end

  for $\forall t, t'$ **do**

    Compute normalized correlations $\zeta_q^{t',t} = r_q^{t',t}/r_q^{t',t'}$

  end

end

Supplementary Table 1 describes a simple train/test split, but it is easy to generalize this procedure to run within a k-fold cross-validation scheme (and then average the

normalized correlations across test folds). In the current study, we employed 10-fold cross-validation.

Fixed interaction structure control

The analysis described in Supplementary Table 1 was designed to be sensitive only to the column spaces of the $\mathbf{W}_x$ and $\mathbf{W}_y$ matrices. To empirically test that our analysis is insensitive to changes in the remaining pCCA model parameters (i.e., that the changes reported in Fig. 6 are solely due to changes to $\mathbf{W}_x$ and $\mathbf{W}_y$), we devised a control based on the following intuition: if we analyze data where the across-area component is held fixed while the within-area component changes, our method (if it works as we expect it to) should indicate that there is no change in interaction between areas. In other words, if we keep the column spaces constant across epochs, we should find that all normalized correlations will be close to 1 (i.e., we identify the same column spaces throughout the trial). To carry out this control analysis, we generated surrogate data that was as similar as possible to the observed activity (in terms of the first and second order statistics, number of trials and number of observed neurons), but with fixed column spaces for the mapping matrices $\mathbf{W}_x$ and $\mathbf{W}_y$.

To achieve this, we first fit a pCCA model to the recorded neural activity, across all epochs, obtaining matrices $\mathbf{W}_x$ and $\mathbf{W}_y$. We then choose matrices $\hat{\mathbf{\Psi}}^t_x$ and $\hat{\mathbf{\Psi}}^t_y$ for each epoch such that $\mathbf{W}_x{\mathbf{W}_x}^T + \hat{\mathbf{\Psi}}^t_x \approx \mathbf{\Sigma}^t_{xx}$ and $\mathbf{W}_y{\mathbf{W}_y}^T + \hat{\mathbf{\Psi}}^t_y \approx \mathbf{\Sigma}^t_{yy}$ for each epoch t. Note that $\mathbf{W}_x$ and $\mathbf{W}_y$ are the same for all epochs. Supplementary Figure 13 illustrates this for two epochs t and t'.

For $\hat{\mathbf{\Psi}}^t_x = \mathbf{\Sigma}^t_{xx} - \mathbf{W}_x{\mathbf{W}_x}^T$ to be a proper covariance matrix, it must be positive definite, which is not guaranteed to be the case (similarly for $\hat{\mathbf{\Psi}}^t_y$). A simple way to ensure that $\mathbf{\Psi}$ is

positive definite is to scale $\mathbf{W}_x$ and $\mathbf{W}_y$ appropriately for each time step, as this operation does not change their column spaces. Supplementary Table 2 describes the surrogate data generation process in detail.

We found that fixing the column spaces of the $\mathbf{W}_x$ and $\mathbf{W}_y$ in this way led pCCA to identify fixed columns spaces across all epochs (Supplementary Fig. 6), indicating the results in Fig. 6 are not driven by changes in the within-area components but rather by changes in the inter-areal interaction structure.

Supplementary Table 2: Creating surrogate data with a fixed interaction structure

Result: Surrogate data $\{\hat{\mathbf{X}}^t, \hat{\mathbf{Y}}^t\}$, for each epoch t and for all choices of q

Given the sets of observations, $\{\mathbf{X}^t, \mathbf{Y}^t\}$, for each trial epoch t

for $q = 1, ..., \min(p_x, p_y)$ **do**

Fit pCCA with latent dim. q jointly to all sets of observations, yielding $\mathbf{W}_x$ and $\mathbf{W}_y$

for $\forall t$ **do**

1. Fit pCCA with latent dim. q to $\{\mathbf{X}^t, \mathbf{Y}^t\}$, yielding $\theta^t = \{\mathbf{W}_x^t, \mathbf{W}_y^t, \mathbf{\Psi}_x^t, \mathbf{\Psi}_y^t\}$
2. Compute minimum within-area variances ${\sigma_{min}}_x^2$ and ${\sigma_{min}}_y^2$, given by the smallest eigenvalues of $\mathbf{\Psi}_x^t$ and $\mathbf{\Psi}_y^t$, respectively
3. Compute across-area variance ratio, defined as the total across-area variance divided by the total variance in each area[1]:
$\nu_x^t = \text{trace}\left(\mathbf{W}_x^t {\mathbf{W}_x^t}^T\right) / \text{trace}\left(\mathbf{W}_x^t {\mathbf{W}_x^t}^T + \mathbf{\Psi}_x^t\right)$ and
$\nu_y^t = \text{trace}\left(\mathbf{W}_y^t {\mathbf{W}_y^t}^T\right) / \text{trace}\left(\mathbf{W}_y^t {\mathbf{W}_y^t}^T + \mathbf{\Psi}_y^t\right)$
4. Scale $\mathbf{W}_x$ and $\mathbf{W}_y$ such that the across-area variance ratios for epoch t are ν_x and ν_y, i.e., choose α_x^t and α_y^t such that:
$\text{trace}\left({\alpha_x^t}^2 \mathbf{W}_x {\mathbf{W}_x}^T\right) / \text{trace}\left({\alpha_x^t}^2 \mathbf{W}_x {\mathbf{W}_x}^T + \mathbf{\Psi}_x^t\right) = \nu_x^t$ and
$\text{trace}\left({\alpha_y^t}^2 \mathbf{W}_y {\mathbf{W}_y}^T\right) / \text{trace}\left({\alpha_y^t}^2 \mathbf{W}_y {\mathbf{W}_y}^T + \mathbf{\Psi}_y^t\right) = \nu_y^t$
5. Compute $\hat{\mathbf{\Psi}}_x^t = \mathbf{\Sigma}_{xx}^t - {\alpha_x^t}^2 \mathbf{W}_x {\mathbf{W}_x}^T$ and $\hat{\mathbf{\Psi}}_y^t = \mathbf{\Sigma}_{yy}^t - {\alpha_y^t}^2 \mathbf{W}_y {\mathbf{W}_y}^T$
6. Using the eigenvalue decompositions of $\hat{\mathbf{\Psi}}_x^t$ and $\hat{\mathbf{\Psi}}_y^t$, set their minimum variance to ${\sigma_{min}}_x^2$ and ${\sigma_{min}}_y^2$, respectively[2]
7. Generate surrogate data $\{\hat{\mathbf{X}}^t, \hat{\mathbf{Y}}^t\}$ from a pCCA model with parameters $\theta^t = \{{\alpha_x^t}^2 \mathbf{W}_x, {\alpha_y^t}^2 \mathbf{W}_y, \hat{\mathbf{\Psi}}_x^t, \hat{\mathbf{\Psi}}_y^t\}$, with the same number of samples as in the entire set of observations, $\{\mathbf{X}^t, \mathbf{Y}^t\}$

end

end

[2]The scale of the estimated mapping matrices is underdetermined (see "Relationship between CCA and pCCA" section below), so this ratio is underdetermined as well. As an example, scaling $\mathbf{W}_x$ by c and $\mathbf{W}_y$ by $1/c$ (thereby changing ν_x and ν_y) results in an equivalent model, from a data likelihood perspective (provided $\mathbf{\Psi}_x$ and $\mathbf{\Psi}_y$ remain positive definite). Although we found that keeping the ratio fixed led to good approximations to the covariance matrices $\mathbf{\Sigma}_{xx}^t$ and $\mathbf{\Sigma}_{yy}^t$, this ratio should not be over-interpreted.

[2]This step is required to ensure that $\hat{\mathbf{\Psi}}_x^t$ and $\hat{\mathbf{\Psi}}_y^t$ are positive definite matrices.

Relationship between CCA and pCCA

The correspondence between the classical formulation and the probabilistic variant of CCA was developed by Bach and Jordan[21]. Specifically, they showed that any maximum likelihood solution derived using the probabilistic model (equations 1-3) corresponds to the same set of canonical dimensions identified using classical CCA. In other words, the data likelihood function for pCCA has infinitely many global optima, where all local optima are also global optima, and all global optima correspond to the same set of canonical dimensions. In particular, if we define the top q canonical dimensions identified for each area by classical CCA as $\mathbf{A}_q$ (a $p_x \times q$ matrix) and $\mathbf{B}_q$ (a $p_y \times q$ matrix), the relationship between the canonical dimensions and the linear mapping matrices from pCCA is given by:

$$\mathbf{W}_x = \mathbf{\Sigma}_{xx}\mathbf{A}_q\mathbf{M}_x \tag{10}$$

$$\mathbf{W}_y = \mathbf{\Sigma}_{yy}\mathbf{B}_q\mathbf{M}_y \tag{11}$$

where $\mathbf{M}_x$ and $\mathbf{M}_y$ are arbitrary $q \times q$ matrices such that $\mathbf{M}_x{\mathbf{M}_y}^T = \mathbf{P}_q$ and the spectral norms of $\mathbf{M}_x$ and $\mathbf{M}_y$ are smaller than one. $\mathbf{P}_q$ is a diagonal matrix containing the first q canonical correlations. As an example, $\mathbf{M}_x = \mathbf{M}_y = \mathbf{P}_q^{1/2}$ satisfies these constraints. Any suitable choice of $\mathbf{M}_x$ and $\mathbf{M}_y$ corresponds to a global maximum of the data likelihood. The link between CCA and pCCA is similar to that between PCA and pPCA, and the derivation of this connection largely follows that originally developed for PCA and pPCA[22,23].

In particular, the fact that $\mathbf{M}_x$ and $\mathbf{M}_y$ are underdetermined means that $\mathbf{W}_x$ and $\mathbf{W}_y$ are not uniquely defined when fitting pCCA, i.e., there are many choices of $\mathbf{W}_x$ and $\mathbf{W}_y$ that result in the same canonical dimensions, and maximizing the data likelihood can return

any such choices. Importantly, these $\mathbf{W}_x$ (resp. $\mathbf{W}_y$) matrices all have the same column space, i.e., multiplication by $\mathbf{M}_x$ (resp. $\mathbf{M}_y$) does not change the column space of $\mathbf{W}_x$ (resp. $\mathbf{W}_y$).

Given two matrices $\mathbf{W}_x$ and $\mathbf{W}_y$ found by maximizing the data likelihood, we cannot directly compute $\mathbf{A}_q$ and $\mathbf{B}_q$ from these matrices alone, since we don't know which $\mathbf{M}_x$ and $\mathbf{M}_y$ the particular solution we found corresponds to. However, since all the consistent $\mathbf{W}_x$ (resp. $\mathbf{W}_y$) matrices have the same column space, we can find the column spaces of $\mathbf{A}_q$ and $\mathbf{B}_q$ by computing matrices $\bar{\mathbf{A}}_q$ and $\bar{\mathbf{B}}_q$ (equations 4 and 6), as the column space of $\bar{\mathbf{A}}_q$ (resp. $\bar{\mathbf{B}}_q$) is the same as the column space of $\mathbf{A}_q$ (resp. $\mathbf{B}_q$) (see Lemma 1 below). Note that the column space of $\mathbf{A}_q$ (resp. $\mathbf{B}_q$) is the subspace of $\mathbf{x}$ (resp. $\mathbf{y}$) spanned by the canonical dimensions found by classical CCA. The relationship above indicates that the subspace spanned by the canonical dimensions in $\mathbf{A}_q$ (resp. $\mathbf{B}_q$) depends on the column space of $\mathbf{W}_x$ (resp. $\mathbf{W}_y$) and on $\mathbf{\Sigma}_{xx}$ (resp. $\mathbf{\Sigma}_{yy}$). In particular, if $\mathbf{\Sigma}_{xx}$ (resp. $\mathbf{\Sigma}_{yy}$) is held fixed, the column space of $\mathbf{A}_q$ (resp. $\mathbf{B}_q$) is solely determined by the column space of $\mathbf{W}_x$ (resp. $\mathbf{W}_y$; see Lemma 2 below). This observation forms the basis for Supplementary Table 1, where we ask how well a pCCA model fit to epoch t (yielding $\mathbf{W}_x^t$ and $\mathbf{W}_y^t$) captures correlations at epoch t'.

Lemma 1. *$\bar{\mathbf{A}}_q$ ($\bar{\mathbf{B}}_q$) and $\mathbf{A}_q$ (resp. $\mathbf{B}_q$) have the same column space.*

Proof. We will show that $\bar{\mathbf{A}}_q$ and $\mathbf{A}_q$ have the same column space. The proof for $\bar{\mathbf{B}}_q$ and $\mathbf{B}_q$ is identical. Starting with equation 10:

$$\mathbf{W}_x = \mathbf{\Sigma}_{xx}\mathbf{A}_q\mathbf{M}_x$$

$$\Leftrightarrow \mathbf{\Sigma}_{xx}^{-1}\mathbf{W}_x = \mathbf{A}_q\mathbf{M}_x$$

$$\Leftrightarrow \bar{\mathbf{A}}_q = \mathbf{A}_q\mathbf{M}_x$$

where we used the fact that $\mathbf{\Sigma}_{xx}$ is a square positive definite matrix. So long as $\mathbf{M}_x$ is a full rank matrix (i.e., the first q canonical correlations are non-zero), $\bar{\mathbf{A}}_q = \mathbf{A}_q\mathbf{M}_x$ and $\mathbf{A}_q$ have the same column space. □

Lemma 2. *If $\mathbf{\Sigma}_{xx}$ ($\mathbf{\Sigma}_{yy}$) is held fixed, the column space of $\mathbf{A}_q$ (resp. $\mathbf{B}_q$) is solely determined by the column space of $\mathbf{W}_x$ (resp. $\mathbf{W}_y$).*

Proof. We will show that the column space of $\mathbf{A}_q$ depends solely on the column space of $\mathbf{W}_x$ if $\mathbf{\Sigma}_{xx}$ is held fixed. The proof for $\mathbf{B}_q$ is identical. Using the compact singular value decomposition $\mathbf{W}_x = \mathbf{UDV}^T$, and inserting it into equation 10:

$$\begin{aligned} &\mathbf{\Sigma}_{xx}\mathbf{A}_q\mathbf{M}_x = \mathbf{W}_x \\ \Leftrightarrow\, &\mathbf{\Sigma}_{xx}\mathbf{A}_q\mathbf{M}_x = \mathbf{UDV}^T \\ \Leftrightarrow\, &\mathbf{A}_q\mathbf{M}_x = \mathbf{\Sigma}_{xx}^{-1}\mathbf{UDV}^T \\ \Leftrightarrow\, &\mathbf{A}_q\mathbf{M}_x\mathbf{VD}^{-1} = \mathbf{\Sigma}_{xx}^{-1}\mathbf{U} \end{aligned}$$

where we used the fact that $\mathbf{\Sigma}_{xx}$ is a square positive definite matrix. As long as $\mathbf{M}_x$ is a full rank matrix (i.e., the first q canonical correlations are non-zero), $\mathbf{M}_x\mathbf{VD}^{-1}$ is a square full rank matrix, and thus $\mathbf{A}_q\mathbf{M}_x\mathbf{VD}^{-1}$ and $\mathbf{A}_q$ have the same column space. So as long as $\mathbf{\Sigma}_{xx}$ is held fixed, the column space of $\mathbf{A}_q$ only depends on $\mathbf{U}$, which is a basis for the column space of $\mathbf{W}_x$. In other words, if we change $\mathbf{W}_x$, only the changes to $\mathbf{U}$ (its column space), and not changes to $\mathbf{D}$ or $\mathbf{V}$, affect the column space of $\mathbf{A}_q$. □

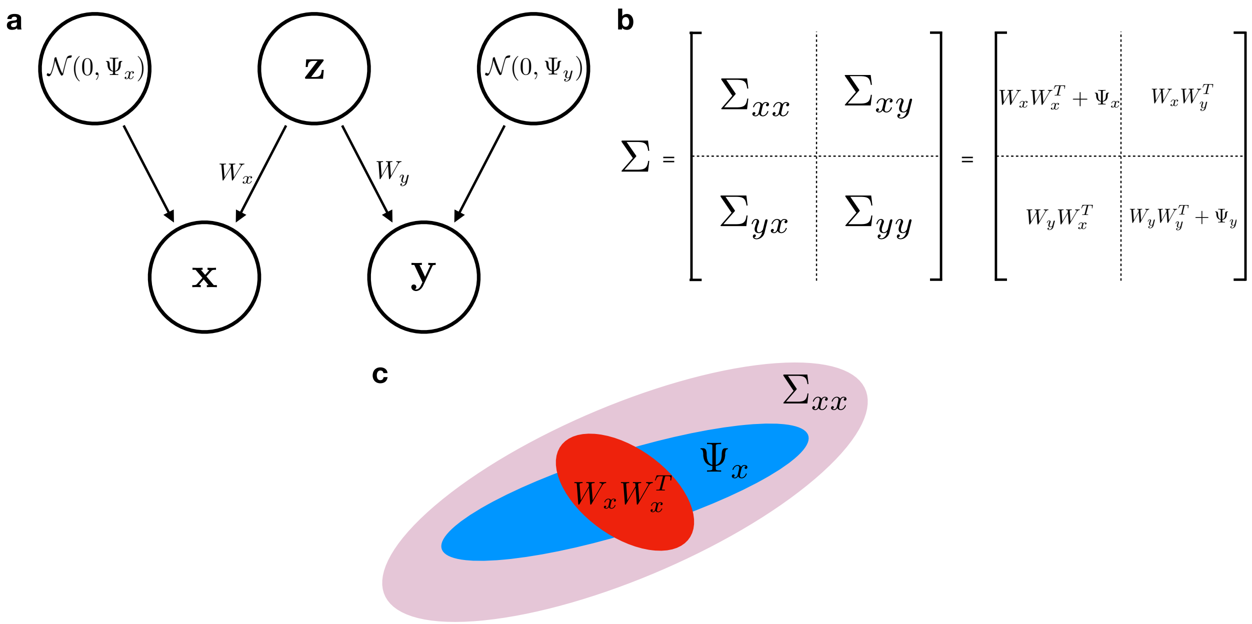

Supplementary Figure 12 Probabilistic canonical correlation analysis (pCCA) **(a)** pCCA's probabilistic graphical model. **(b)** Summary of the relationship between the data covariance matrices and the pCCA model parameters. **(c)** Graphical representation of the covariance decomposition under a pCCA model for one of the two populations. Red ellipse represents the across-area component; blue ellipse represents the within-area component; pink ellipse represents the total covariance in this area.

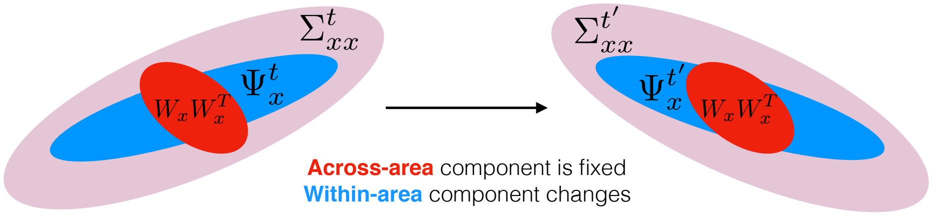

Supplementary Figure 13 Changing the total covariance in one of the areas while keeping the across-area component fixed. Same conventions as in Supplementary Fig. 12c.

Supplementary References

1. Smith, M. A. & Kohn, A. Spatial and Temporal Scales of Neuronal Correlation in Primary Visual Cortex. *The Journal of Neuroscience* **28**, 12591–12603 (2008).

2. Williams, P. E., Mechler, F., Gordon, J., Shapley, R. & Hawken, M. J. Entrainment to video displays in primary visual cortex of macaque and humans. *Journal of Neuroscience* **24**, 8278–8288 (2004).

3. Jia, X., Tanabe, S. & Kohn, A. Gamma and the Coordination of Spiking Activity in Early Visual Cortex. *Neuron* **77**, 762–774 (2013).

4. Jia, X., Xing, D. & Kohn, A. No consistent relationship between gamma power and peak frequency in macaque primary visual cortex. *Journal of Neuroscience* **33**, 17–25 (2013).

5. Zandvakili, A. & Kohn, A. Coordinated Neuronal Activity Enhances Corticocortical Communication. *Neuron* **87**, 827–839 (2015).

6. Reich, D. S., Mechler, F. & Victor, J. D. Independent and redundant information in nearby cortical neurons. *Science* **294**, 2566–2568 (2001).

7. Bair, W., Zohary, E. & Newsome, W. T. Correlated firing in macaque visual area mt: time scales and relationship to behavior. *Journal of Neuroscience* **21**, 1676–1697 (2001).

8. Gregoriou, G. G., Gotts, S. J., Zhou, H. & Desimone, R. High-Frequency, Long-Range Coupling Between Prefrontal and Visual Cortex During Attention. *Science* **324**, 1207–1210 (2009).

9. Salazar, R. F., Dotson, N. M., Bressler, S. L. & Gray, C. M. Content-Specific Fronto-Parietal Synchronization During Visual Working Memory. *Science* **338**, 1097–1100 (2012).

10. van Kerkoerle, T. *et al.* Alpha and gamma oscillations characterize feedback and feedforward processing in monkey visual cortex. *Proceedings of the National Academy of Sciences of the United States of America* **111**, 14332–14341 (2014).

11. Fries, P., Reynolds, J. H., Rorie, A. E. & Desimone, R. Modulation of Oscillatory Neuronal Synchronization by Selective Visual Attention. *Science* **291**, 1560–1563 (2001).

12. Arce-McShane, F. I., Ross, C. F., Takahashi, K., Sessle, B. J. & Hatsopoulos, N. G. Primary motor and sensory cortical areas communicate via spatiotemporally coordinated networks at multiple frequencies. *Proceedings of the National Academy of Sciences* **113**, 5083–5088 (2016).

13. Michalareas, G. *et al.* Alpha-beta and gamma rhythms subserve feedback and feedforward influences among human visual cortical areas. *Neuron* **89**, 384–397 (2016).

14. Bastos, A. *et al.* Visual Areas Exert Feedforward and Feedback Influences through Distinct Frequency Channels. *Neuron* **85**, 390–401 (2015).

15. Krishna, A., Tanabe, S. & Kohn, A. Decision signals in the local field potentials of early and mid-level macaque visual cortex. *Cerebral Cortex* **31**, 169–183 (2021).

16. Bokil, H., Andrews, P., Kulkarni, J. E., Mehta, S. & Mitra, P. P. Chronux: a platform for analyzing neural signals. *Journal of neuroscience methods* **192**, 146–151 (2010).

17. Barnett, L. & Seth, A. K. The MVGC multivariate Granger causality toolbox: A new approach to Granger-causal inference. *Journal of Neuroscience Methods* **223**, 50–68 (2014).

18. Bosman, C. *et al.* Attentional Stimulus Selection through Selective Synchronization between Monkey Visual Areas. *Neuron* **75**, 875–888 (2012).

19. Semedo, J. D., Zandvakili, A., Machens, C. K., Yu, B. M. & Kohn, A. Cortical Areas Interact through a Communication Subspace. *Neuron* **102**, 249–259.e4 (2019).

20. Semedo, J. D., Gokcen, E., Machens, C. K., Kohn, A. & Byron, M. Y. Statistical methods for dissecting interactions between brain areas. *Current opinion in neurobiology* **65**, 59–69 (2020).

21. Bach, F. R. & Jordan, M. I. A probabilistic interpretation of canonical correlation analysis (2005).

22. Roweis, S. T. EM algorithms for PCA and SPCA. In *Advances in neural information processing systems*, 626–632 (1998).

23. Tipping, M. E. & Bishop, C. M. Probabilistic Principal Component Analysis. *Journal of the Royal Statistical Society: Series B (Statistical Methodology)* **61**, 611–622 (1999).
